# Prevalence and Risk Factors for Suicidal Ideation Following the COVID-19 Pandemic in Kerman Province: A Cross-Sectional Study

**DOI:** 10.34172/aim.2023.103

**Published:** 2023-12-01

**Authors:** Shiva Pouradeli, Hassan Ahmadinia, Mohsen Rezaeian

**Affiliations:** ^1^Occupational Environment Research Center, Medical School, Rafsanjan University of Medical Sciences, Rafsanjan, Iran; ^2^Clinical Research Development Unit, Shafa Hospital, Kerman University of Medical Sciences, Kerman, Iran; ^3^National Agency for Strategic Research in Medical Sciences Education, Tehran, Iran

**Keywords:** COVID‐19, Cross-sectional study, Iran, Pandemic, Suicidal ideation

## Abstract

**Background:**

Suicidal ideation (SI) serves as an important predictor of suicide. The prevalence of SI has increased following the COVID-19 pandemic. This study aims to investigate the prevalence and risk factors associated with SI after the pandemic in the Kerman province.

**Methods:**

This cross-sectional study was conducted in 23 counties of the Kerman province between 2021 and 2022. The Beck Scale for Suicidal Ideation (BSSI) was utilized to estimate SI, while multiple logistic regression analysis was employed to examine the impact of various variables on SI.

**Results:**

A total of 1421 individuals (47.7% men, 50.0% women and 2.3% unknown) with an average age of 35.17±9.47 years participated in this study. The estimated prevalence rate of SI was 9.2%, with variations ranging from 0% to 42% across different counties. Individuals with SI exhibited a significantly younger mean age and fewer family members. Furthermore, SI was significantly more prevalent among single participants, unemployed individuals, students, those with a history of mental illness, prior psychiatric medication use, and previous SI. Employed individuals had 87% lower odds of experiencing SI compared to the unemployed. Individuals with a history of prior SI had 239 times higher odds of SI than those without such a history. Additionally, each year increase in age corresponded to an 8.8% decrease in the odds of SI.

**Conclusion:**

The high prevalence of SI is concerning, and it is essential to remain vigilant regarding its health and social consequences as the pandemic continues. Therefore, it is imperative to provide enhanced mental health services, particularly targeting at-risk groups.

## Introduction

Suicide is a major public health problem worldwide.^1^ Globally, suicide is the fourth leading cause of death among people under 30 years of age.^2^ Annually, more than 700 000 people die by suicide and there are more than 20 suicide attempts for each suicide.^3^ Most suicides occur in low- and middle-income countries,^4^ where early detection is difficult due to limited resources and services and inadequate treatment and support.^5^ In Iran, the 20-year suicide mortality trend has increased with an estimated mean rate of 9.9 per 100 000 persons.^6^

The coronavirus (COVID-19) pandemic was a health crisis that was not experienced in the last 100 years.^7^ This pandemic had a substantial effect on the global economy and raised the unemployment rate in the world.^8,9^ On the other hand, the measures taken to address the COVID-19 crisis increased mental health challenges, substance use, depression, and anxiety disorder in affected communities.^10^ Suicidal ideation (SI) “(i.e. thinking about, considering, or planning for suicide)”,^11^ increased during this pandemic,^10^ especially among people under lockdown due to the coronavirus.^12^ There is a positive relationship between SI and suicide attempts. Suicidal ideas first appear and intensify later to become permanent and invasive. These ideas lead to the establishment of a suicidal plan and subsequently to death upon the successful execution of the plan. Whenever the outcome of the act is not fatal, it is defined as a suicide attempt.^13^ Thus, SI is an important predictor of suicide.^14^ In two recent surveys in Iran (2021), SI was reported at 8.6% in older adults in Shiraz and 20.8% in Qazvin.^15,16^ The important factors for SI in these studies were depression, being single, inability to pay medical bills, low social support, low social communication, fear of COVID-19, and insomnia.^15,16^

Kerman is the ninth most populous and largest province in Iran. This province is significant in industry, culture, politics, agriculture, higher education, and religion.^17^ The suicide rate increased in the first year of the COVID-19 pandemic in the Kerman province.^18^ Given the lack of information about SI, the negative impact of this pandemic on mental health, and the rise of suicide, this study aims to investigate SI after the COVID-19 pandemic in the Kerman province. Given the large population and cultural diversity in this province, the results of this study can be generalized to the provinces in southeastern Iran.

## Materials and Methods

###  Study Population and Data Collection

 This cross-sectional survey was administered from March 21, 2021 to March 21, 2022 in the Kerman province during the fourth to the sixth COVID-19 waves.

 The study population included people living in the Kerman province. Due to the limitations imposed by COVID-19, house visits and questionnaire gathering were not feasible. Instead, data were collected from individuals visiting the Departments of Cooperatives, Labor, and Social Welfare of 23 counties in the Kerman Province. These departments serve a diverse range of clients, including employers, job seekers aged 15 and above, those seeking support with insurance-related matters, individuals filing workplace complaints or reporting work-related accidents, and individuals verifying their educational qualifications for employment. The inclusion criteria specified residency in the city where data was collected and providing informed consent. Individuals who were unable to write or had limited literacy were interviewed orally, with the interviewer transcribing their responses. The exclusion criterion encompassed individuals lacking the mental ability to respond accurately to the questionnaire and those who failed to answer the questionnaire completely. First, this questionnaire was described for the participants and they declared their informed consent to participate in the study. Then, the questionnaires were completed by the volunteers. A two-part questionnaire was used to collect the data. The first part included questions about the participants’ demographic characteristics and mental health, and the second part was the Beck Scale for Suicidal Ideation (BSSI).

###  Sample Size

In a previous study conducted in two counties of the Kerman province (2009), SI was reported to be 10%.^19^ The sample size was calculated using Cochran’s formula. Then, the sample size for each county was calculated as follows:

*ni = (Ni ⁄N) × n*

Where*ni* is the sample size for each county; *Ni* is the population of i-th county based on the 2016 census in Iran, *N* is the total population of the province based on the 2016 census in Iran, and *n* is the sample size estimated using Cochran’s formula. Finally, 1498 persons were considered as the sample size. The participants were selected using proportional sampling from 23 counties of the Kerman province (Table 1).

**Table 1:** Sample Size and Response Rate for Each County

**County**	**Population - 2016 Census**	**Sample Size**	**Participants**	**Response Rate (%)**
Kerman	738724	350	350	100
Sirjan	324103	154	142	92
Rafsanjan	311214	148	136	92
Jiroft	308858	146	135	92
Bam	228241	108	100	93
Zarand	138133	65	61	94
Rudbar-e Jonubi	105992	50	47	94
Shahr Babak	103975	49	46	94
Kahnooj	95848	45	42	93
Rigan	88410	42	39	93
Baft	84103	40	40	100
Anbarabad	82438	39	36	92
Bardsir	81983	39	36	92
Qaleh Ganj	76495	36	34	94
Fahraj	67096	32	30	94
Manujan	65705	31	29	94
Narmashir	54228	26	24	92
Ravar	43198	20	19	95
Orzoeiyeh	38510	18	17	94
Anar	36897	17	17	100
Rabor	35362	17	16	95
Faryab	34000	16	15	94
Kuhbanan	21205	10	10	100
Total	3164718	1498	1421	95

###  Instruments

*BSSI:* The BSSI was invented by Aaron Beck in 1961. This scale has 19 items to measure the current intensity of patients’ specific attitudes, behaviors, and plans to carry out suicide. Each question has three options showing suicidal intensity scored on a 3-point scale ranging from 0 to 2. The total score ranges from 0 to 38 as follows: 0–3 = no suicidal ideation; 4–11 = low-risk suicidal ideation; and 12–38 = high-risk suicidal ideation. The BSSI contains five screening questions. Three questions assess the wish to live or the wish to die, and two items assess the desire to attempt suicide. If the respondent reports any active or passive desire to carry out suicide, then 14 additional questions are administered. The BSSI takes approximately 10 minutes to complete. The validity and reliability of the English version of BSSI have been repeatedly assessed and the Cronbach’s alpha coefficient has always been higher than 0.85. The validity and reliability of this questionnaire have been confirmed in Iran.^20-22^

###  Statistical Analysis

 The data were summarized using descriptive statistics including frequency (percentage, 95% confidence interval) for qualitative variables and mean ± standard deviation for quantitative variables. The prevalence of SI was calculated for each county and the whole province. The geographical distribution of the prevalence of SI on the map for each county was prepared using the ArcGIS 10.3 software.

An independent samples t-test was run to compare the quantitative variables between people with and without SI. A chi-square test was used to compare SI in terms of the qualitative variables. Multiple logistic regression analysis was also used to investigate the impact of variables on SI. The dependent variable was SI. The independent variables were other demographic and mental health variables. The enter method was used to perform this test. The first group is defined as a reference categorical covariate. IBM-SPSS (version 24) was used for data analysis. The *P* value of 0.05 was considered as the significance level.

## Results

**Figure 1:**
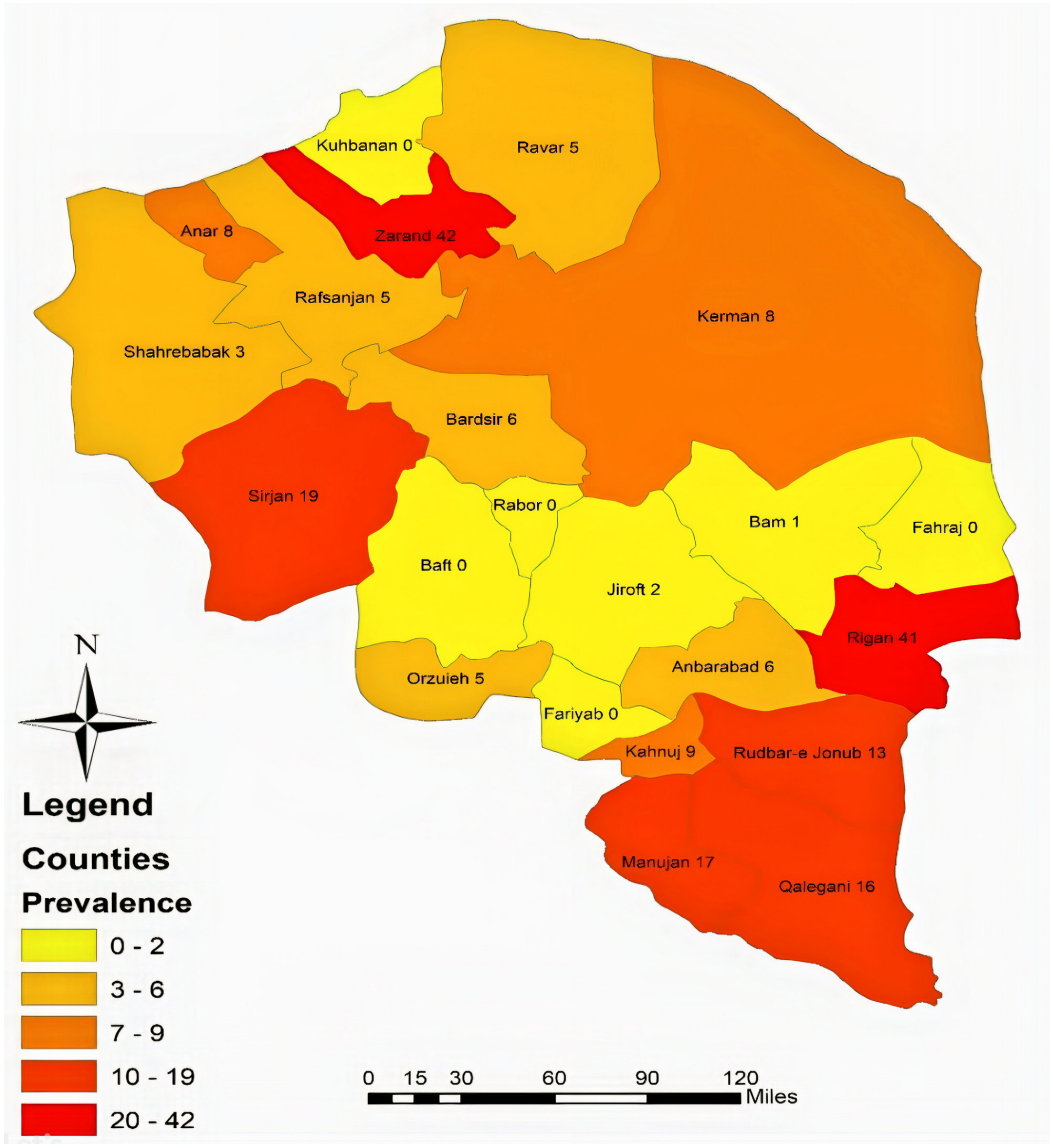
Prevalence of Suicidal Ideation in the Counties of the Kerman Province

A total of 1421 persons out of 1498 participants completed the questionnaire. The response rate was 95% (Table 1). The prevalence of SI was estimated at 9.2% (95% CI: 7.71%–10.72%) in the Kerman province. Sixty-three persons (4.4%) were in the low-risk group, and 68 persons (4.8%) were in the high-risk group for suicide. The prevalence of SI in the counties varied from 0 to 42%. The prevalence of SI was less than 2% in Baft, Rabor, Faryab, Fahraj, Kuhbanan, Narmashir, Bam, and Jiroft. It varied from 3% to 9% in Shahr Babak, Rafsanjan, Orzoeiyeh, Ravar, Bardsir, Anbarabad, Kerman, Anar, and Kahnooj. It was between 10%-20% in Rudbar-e Jonubi, Qaleh Ganj, Manujan, and Sirjan and around 40% in Rigan and Zarand (Figure 1).

 The data showed that 710 participants were men and 678 participants were women with an average age of 35.17 ± 9.47 years (range, 13-71 years). Moreover, 893 of them (63%) were married and 423 persons (30%) were single. A total of 25 persons (2%) were illiterate, 33% had diploma or under diploma education and 62% had a higher education. The results also showed that 735 individuals (52%) were employed, 368 persons (26%) were unemployed, and 161 persons (11%) were housewives. In addition, 602 persons (42%) were the heads of the family, who had an average of 3 ± 1 (range, 1-8) people under their care.

There was no statistically significant difference in the number of men and women with SI (*P* = 0.572). There was no significant difference in the proportion of people with SI who had different education levels (*P* = 0.506). Moreover, there was no statistically significant difference in the proportion of people with SI who were or were not the head of the family (*P* = 0.995).

However, the SI prevalence differed significantly in the participants in terms of marital status (*P* = 0.012), occupation (*P* < 0.001), history of mental illness (*P* < 0.001), history of psychiatric medication use (*P* < 0.001), and history of prior SI (*P* < 0.001). The prevalence of SI was significantly higher in single, unemployed persons and students compared to other job groups. In addition, the prevalence of SI was significantly higher in people with a history of mental illness, a history of psychiatric medication use, and a history of prior SI (Table 2).

**Table 2:** Prevalence of SI in Terms of Demographic Characteristics and Mental Health

**Variable**	**Category**	**Total**	**Suicidal ideation**		***P*** ** Value**
			**Yes**		
		**No. (%)**	**No. (%)**	**95% CI**	
Gender	Male	710 (50.0%)	68 (9.6%)	(7.4– 11.8)	0.572
	Female	678 (47.7%)	59 (8.7%)	(6.6– 10.8)	
	Unknown	33 (2.3%)	4 (12.1%)	(1.0– 23.2)	
Marital status	Single	423 (29.8%)	53 (12.5%)	(9.4-15.7)	0.012
	Married	893 (62.8%)	67 (7.5%)	(5.8-9.2)	
	Divorced	64 (4.5%)	7 (10.9%)	(3.3-18.6)	
	Unknown	41 (2.9%)	4 (9.8%)	(0.7-18.8)	
Education	Illiterate	25 (1.8%)	3 (12.0%)	(-0.7-24.7)	0.506
	Diploma or under diploma	473 (33.3%)	48 (10.1%)	(7.4-12.9)	
	Higher education	878 (61.8%)	74 (8.4%)	(6.6-10.3)	
	Unknown	45 (3.2%)	6 (13.3%)	(3.4-23.3)	
Occupation	Student	60 (4.2%)	7 (11.7%)	(3.5-19.8)	< 0.001
	Employed	735 (51.7%)	41 (5.6%)	(3.9-7.2)	
	Housewife	161 (11.3%)	10 (6.2%)	(2.5-9.9)	
	Unemployment	368 (25.9%)	60 (16.3%)	(12.5-20.1)	
	Retired	7 (0.5%)	0 (0.0)	(0)	
	Unknown	90 (6.3%)	13 (14.4%)	(7.2-21.7)	
Head of the family	Yes	602 (42.4%)	55 (9.1%)	(6.8-11.4)	0.995
	No	778 (54.8%)	71 (9.1%)	(7.1-11.1)	
	Unknown	41 (2.9%)	5 (12.2%)	(2.2-22.2)	
History of mental illness	Yes	36 (2.5%)	17 (47.2%)	(30.9-63.5)	< 0.001
	No	1072 (75.4%)	68 (6.3%)	(4.9-7.8)	
	Unknown	313 (22.0%)	46 (14.7%)	(10.8-18.6)	
History of psychiatric medication use	Yes	27 (1.9%)	14 (51.9%)	(33.0-70.7)	< 0.001
	No	1077 (75.8%)	68 (6.3%)	(4.9-7.8)	
	Unknown	317 (22.3%)	49 (15.5%)	(11.5-19.4)	
History of suicidal ideation	Yes	33 (2.3%)	25 (75.8%)	(61.1-90.4)	< 0.001
	No	1066 (75.0%)	59 (5.5%)	(4.2-6.9)	
	Unknown	322 (22.7%)	47 (14.6%)	(10.7 – 18.5)	

The mean age of people with SI was significantly lower than people without SI (*P* = 0.004). The people with SI were usually from families with a significantly smaller number of members compared to people without SI (*P* = 0.028) (Table 3).

**Table 3:** Prevalence of SI in Terms of Age and Family Size

	**Total Number**	**Suicidal ideation**						***P*** ** value**
		**Yes**			**No**			
		**Number**	**Mean**	**SD**	**Number**	**Mean**	**SD**	
Age	1334	122	32.83	9.149	1212	35.40	9.473	0.004
Number of family members	535	39	2.41	1.093	496	2.84	1.192	0.028

Based on multiple logistic regression, the odds of SI in the employed persons were 87% lower than in the unemployed persons (OR = 0.13; CI 95%: 0.029-0.578; *P* = 0.007). The odds of SI in the individuals with a history of prior SI were 239 times more than in the individuals without a history of prior SI (OR = 239.995; CI 95%: 18.113-3179.880; *P* < 0.001). With each year of increase in age, the odds of having SI diminished by 8.8% (OR: 0. 912, CI 95%: 0.833–0.997; *P* = 0.044). The effect of other variables on the chance of having SI was not statistically significant (*P* > 0.05) (Table 4).

**Table 4:** Impact of Demographic and Mental Health Variables on SI—Using Multiple Logistic Regression

**Variable**	**Category**	**Exp(B)**	**95% CI for EXP(B)**		***P*** ** value**
			**Lower**	**Upper**	
Age	-	0.912	0.833	0.997	0.044
Gender	Male Female^*^	0.130	0.008	2.136	0.153
Marital status	Other Single^*^	1.890	.319	11.182	0.483
Education Level	Diploma or Under diploma	0.618	0.010	37.369	0.818
	Bachelor’s or undergraduate degree	0.389	0.037	4.138	0.434
	Master’s degree	0.142	0.012	1.639	0.118
	Illiterate^*^				
Occupation	Housewife	1.472	0.086	25.233	0.790
	Employed	0.130	0.029	.578	0.007
	Student	5.032	0.208	121.634	0.320
	Unemployed^*^				
History of mental illness	No	1.887	0.134	26.563	0.638
	Yes^*^				
History of suicidal ideation	No	239.995	18.113	3179.880	< 0.001
	Yes^*^				
Number of family members		0.874	0.501	1.525	0.636
Constant		14.005			0.170

*Reference group.

## Discussion

 The overall prevalence of SI was estimated at 9.2% in this study. It varied from 0% to 42% among the counties of the Kerman province. The prevalence of SI was less than 2% in 8 counties, 3% to 9% in 9 counties, 10% to 20% in 5 counties, and about 40% in 2 counties. These data indicate that the prevalence of SI is higher in the south, except for two counties, moderate in the north, and low in the center of the province.

Due to its large population and vastness, the Kerman province has a unique position for its industrial centers, industries, cultural norms, socioeconomic factors, agricultural activities, and higher education institutions.^17^ Cultural factors like family conflict and acceptability of suicide are related to suicide ideation.^23^ Hence, culture influences how SI, intentions, plans, and attempts are expressed.^24^ Besides, socioeconomic factors influence mental health outcomes, and low income is a risk factor for SI and attempts.^25^ Therefore, in areas with similar cultural and socio-economic backgrounds, the prevalence of SI is almost in the same range.

The prevalence of SI is high in the southern part of the province because one-third of deprived areas are located in this part of the province. Ten cities in the south and east of the Kerman province are deprived of any amenities. Poverty and deprivation in the southern and eastern areas account for many social harms.^26^

The prevalence of SI is high in the northern areas of the province such as Sirjan and Zarand. A previous study showed that the rate of suicide attempts was high in Sirjan perhaps due to the geographical status of the city (easy access to drugs, opium, and agricultural pesticides) and hard work conditions in mines.^27^ Other studies have also shown that the incidence of suicide in Zarand is much higher than the average global, national, and provincial rate.^28,29^ Therefore, it seems that suicide is an unsolved problem in this county that needs serious interventions.

The Kerman Province has undergone many developments since 50 years ago due to its various industries, and its population has increased by nine times during this period. Cities have faced many problems because of rapid urban growth without developing economic and social indicators.^30^ This can account for the high prevalence of SI in some developing counties in the province.

Navadeh et almeasured the prevalence of SI using the Scale for Suicide Ideation (SSI) and reported it at 10% in people aged 15 and older in Bardsir and Mahan. Moreover, the prevalence of SI was 7.33% in non-suicide attempters and 48.3% in suicide attempters.^19^ Mahan is a small city in the Kerman County with a population of 19 423 persons.^31^ The prevalence of SI in the present study was 6% and 8% in the Bardsir and Kerman Counties, respectively. It was 75.8% in individuals with prior SI and 5.5% in individuals without prior SI. A comparison of the prevalence of SI between suicide attempters and individuals with prior SI indicated that SI was much higher among suicide attempters. SI was also higher in individuals whit prior SI compared to non-suicide attempters. A systematic review study confirmed a higher prevalence of SI after the COVID-19 pandemic.^32^ However, the sampling method and SI instruments were different in these two studies. Therefore, these issues should be considered for a more accurate comparison of SI before and after the COVID-19 pandemic. Furthermore, the prevalence of SI was unknown in other counties before the pandemic, so we could not compare the results of the present study with the data reported in previous studies.

Various prevalence rates of SI were reported in different cities and countries during the COVID-19 pandemic. It was 16.4% in Chinese adults aged 18 years and older using the Patient Health Questionnaire-9 (PHQ-9) and convenience sampling method.^33^ It was 16.2% using PHQ-9 in Australian adults, who had direct experience with COVID-19 during the eighth wave of the COVID-19 pandemic.^33^ It was 12% among Canadian adults (16 years and older) based on self-reporting any thought of suicide.^11^ It was 8.8% in the Spanish population aged 18 years or over using the Spanish version of the Depression, Anxiety, and Stress Scale (DASS-21).^34^ It was 5.20% in the Greek population base on a web-based survey, using the Patient Health Questionnaire (PHQ-2), and snowball sampling.^35^ A systematic review study showed that the pooled prevalence of SI was 12.1% in 12 studies.^32^ In addition, the prevalence of SI was 20.4% in psychiatric patients during the COVID-19 pandemic as indicated by a meta-analysis.^36^ Two recent surveys in Iran (2021) reported that the prevalence of SI was 20.8% among adults aged over 18 years in Qazvin using PHQ-9, and 8.6% in older adults aged over 60 years in Shiraz (using the Suicide-Screening Questionnaire (ASQ)).^15,16^ Shiraz and Kerman are located in southwestern Iran and have many social and cultural similarities. The prevalence of SI in the two areas does not differ much. However, to compare the prevalence of SI in Kerman with other areas, it is necessary to pay attention to the differences in measurement tools, data collection methods, and the participants’ age. It also should be noted that sociocultural and socioeconomic factors, demographic characteristics, and cultural and moral values can affect SI.^37^

In the present study, the age of people with SI was lower than that of people without SI. It means that younger people are more likely to have SI, as indicated in other studies.^38,39^

In line with a study by Shiraly et al, the data in the present study indicated that SI was statically higher in single people.^16^ In contrast, Lin et al and Khajedaluee et al reported a higher prevalence of SI among married people.^14,15^ This difference could be attributed to different lifestyles in different regions.

Unemployed individuals and students have more SI compared to employed people. Similarly, a study in China found that unemployment was associated with an increased risk of SI.^33^ Moreover, men who had lost their jobs due to the COVID-19 pandemic were twice more likely to have SI.^40^ Thus, unemployment and job loss have a significant correlation with higher levels of SI.^41^

The rate of SI was twice as high in German students during the COVID-19 pandemic compared to previous years.^42^ Besides, in the Rafsenjan county, Kerman province, SI among paramedical students was relatively high before the pandemic.^43^ However, a systematic review study showed that SI was elevated in college students during the COVID-19 pandemic, and SI could increase or decrease considerably over the days.^44^

In the present study, the prevalence of SI was higher in people with a history of mental illness, a history of psychiatric medication use, and a history of prior SI. Previous reports in Iran also showed that SI was significantly more in people with poor to moderate physical health and depression.^16^ Wise confirmed that individuals with pre-existing mental health conditions were more likely to experience SI.^39^ Psychiatric patients and those with poor mental health may be at higher risk of COVID-19.^45^ They were socially and economically vulnerable even before the crisis. These people comprise a significant portion of people who attempted suicide or die worldwide.^7^ Therefore, there is an urgent need to scale up and improve mental health and psychosocial support services, especially for individuals with poor mental health.

 The most important limitation of this study was traveling restrictions and the challenges of data collection due to the COVID-19 pandemic. Due to COVID-19 limitations, house visits and questionnaire gathering were not feasible. Additionally, phone cooperation was also challenging. Therefore, the time to conduct this study was long and samples were selected from those visiting the Departments of Cooperatives, Labor, and Social Welfare. Thus, the prevalence of SI should be assessed with a larger sample of the general population in each county.

## Conclusion

 Suicide ideation is high in the Kerman Province, especially in some counties. The high prevalence of SI is concerning, and we need to remain vigilant about its health and social consequences as the pandemic continues. Therefore, people’s mental health should be promoted by providing a higher level of mental health services, especially for at-risk groups during the COVID-19 pandemic.
